# Analog Resonance Computation: A New Model for Human Cognition

**DOI:** 10.3389/fpsyg.2020.02080

**Published:** 2020-09-09

**Authors:** Aidan J. Byrne

**Affiliations:** Consultant Anaesthetist, Swansea Bay University Health Board, Honorary Professor, Medical School, Swansea University, Swansea, United Kingdom

**Keywords:** cognition, decision making, human error, learning, behavior

## Abstract

Early models of human cognition appeared to posit the brain as a collection of discrete digital computing modules with specific data processing functions. More recent theories such as the Hierarchically Mechanistic Mind characterize the brain as a massive hierarchy of interconnected and adaptive circuits whose primary aim is to reduce entropy. However, studies in high workload/stress situations show that human behavior is often error prone and seemingly irrational. Rather than regarding such behavior to be uncharacteristic, this paper suggest that such “atypical” behavior provides the best information on which to base theories of human cognition. Rather than using a digital paradigm, human cognition should be seen as an analog computer based on resonating circuits whose primary driver is to constantly extract information from the massively complex and rapidly changing world around us to construct an internal model of reality that allows us to rapidly respond to the threats and opportunities.

## Introduction

There is currently no widely accepted model of human cognition which resolves the results of studies in both neuroscience and human psychology (Badcock et al., 2019). In addition, the analysis of real life situations often reveals behavior which does not fit with ideas of human cognition as based on logical decision making. (Example)

During complex surgery, two anesthetists were present, a senior with many years’ experience in that area and an inexperienced anesthetist who had not seen the procedure before. At around 2 h into the procedure, the senior anesthetist, suddenly increased the infusion of intravenous fluid, asked for blood and got it ready to infuse, despite their being no change in the patient’s pulse or blood pressure. The inexperienced anesthetist questioned the decision to give blood, but almost immediately the patient’s blood pressure fell and it became obvious that they were bleeding heavily. Blood transfusion was therefore the correct action and the patient was treated successfully.

When asked, the senior anesthetist could not explain why they had started to treat the patient so quickly. However, after some thought, three factors were identified. Firstly, they knew that 2 h into the procedure was when blood loss was most likely, and that any bleeding at that point was going to be rapid and need immediate treatment. Secondly, the surgeon had asked for an “M11,” which was an old type of heavy duty clip applicator, which was their long standing request when a large blood vessel had been cut and there was rapid bleeding. A different clip applicator had been in use for some time, but the surgical assistant knew what was required when asked for an “M11.” Thirdly, the sound of surgical suction changes when blood flow into the wound increases. The change is very difficult to describe, but easily recognized after years listening to the same procedure.

The learning points were that the anesthetist used three different cues to guide decision making which are not included in any textbook description of how to identify bleeding during surgery and, if asked about their normal practice, they would not have identified them as cues that they used. In addition, the less experienced anesthetist was completely unable to access this information [It can be noted that this problem could have been avoided by the surgeon warning the anesthetist of the bleeding, but intraoperative communication during surgery is known to be problematic (Nagpal et al., 2012)].

Many theories of human cognition are based on digital and computational mechanisms with the brain conceptualized as consisting of modules with specific neural functions turned on or off by relevant cognitive mental activity. This is supported by functional MRI studies which demonstrate increased neuronal activity in response to isolated cognitive tasks completed by subjects in a laboratory setting (Binder et al., 1999). It is also supported by neuroanatomical models which shown that localized damage to specific areas of the brain are associated with specific neurological deficits (Cramer et al., 1997). For example, damage to the occipital area of the cortex leads to loss of vision.

In contrast, studies of cognitive workload often use rapidly changing and complex tasks and posit highly constrained cognitive resources which are easily overwhelmed (Byrne, 2011). For example, research using medical simulators has identified widespread patterns of error and poor performance in those judged by accepted methods as both knowledgeable and competent (Byrne et al., 1998, 2013). In particular, the field of Human Factors research has found that not only is poor performance common in complex environments, but also that major errors in perception and even seemingly bizarre behavior is not uncommon. Further, this “abnormal” behavior is not random, but has well-defined patterns that can be predicted and mitigated by changes to training, task or environment (Reason, 1990).

This inconsistency has led to the concept of two different types of cognition: Type I which is rapid and based on pattern recognition and Type II which is slower and logical/analytical (Kahneman, 2011). This distinction has often led educators to regard Type I thinking as primitive, biased and error prone, with the conclusion that students should be taught to use proper, logical and accurate Type II thinking (Croskerry et al., 2013). However, while this paradigm is plausible and neatly explains many of the problematic areas of human behavior, it has increasingly been questioned (Grayot, 2020), principally because there appears to be little evidence that such systems actually exist within the brain. A fundamental principle used in the construction of this paper is that rather than human error providing inconvenient data that needs to be explained by residual primitive systems or just ignored, error is regarded as providing key insights into how the brain works.

More recent theories such as the Hierarchically Mechanistic Mind (HMM) (Badcock et al., 2019) characterize the brain as a “a complex adaptive system” based on the need to rapidly minimize differences between the internal models of an individual and their sensory input. It posits that the brain consists of a hierarchy of modules, “ranging from lower-order psychobiological mechanisms characterized by automatic, serial processing, and a high degree of specialization, through to higher-level modules that are flexible in their responses to input and production of outputs, allow us to gain awareness of these outputs, and enable top-down cognitive control” (Badcock et al., 2019).

The principle suggestion made here is that HMM provides a good model for cognition, but that in addition, cognition should be seen as an analog rather than digital process. Although the term computer is now almost synonymous with digital technology, the earliest computers such as astrolabes and slide rules used multiple components with mechanical linkages so that data was inputted by moving parts of the mechanism and the result being read from other parts of the mechanism. In the 1950s, much more complex electromechanical devices were developed, such as the Mark IV naval fire control computer designed to control the guns on warships (Ben Clymer, 1993). This device could integrate ship speed/course/roll/yaw/pitch, target distance, ammunition type and even the effect of the earth’s rotation to guide shells accurately on to targets many miles from the ship. Such devices are known to provide extremely rapid and accurate outputs. Although naturally suited to analyzing continuous variables such as speed/distance, analog devices can also produce discrete outputs. For example, a coin flip can be used as an analog yes/no device and a roulette wheel used to select numerical outputs. Despite their simplicity, analog systems can produce highly complex outputs, making them highly resource efficient, a key evolutionary advantage (Allen et al., 2011). However, analog devices were superseded by digital computers possibly because each factor added to the computation produces an exponential rise in the complexity of the device and because any change in the program required the device to be physically rebuilt (Small, 2001). While this became a limiting factor to manufactured analogue computers, the inherent complexity and able to self-reconstruct means that these problems would not limit the use of analog computation by the brain.

While individual neurons are effectively digital in that they are either in a resting state or go through a rapid process of activation and resetting, groups of neurons are arranged in circuits that fire in regular sequences which produce rhythmic electrical activity. This activity can which can be recorded at the scalp as the Electroencephalogram (EEG) which shows characteristic frequency changes with specific mental activity (Golnar-Nik et al., 2019). The suggestion here is that the basic functional cognitive component is a group of neurons which are arranged so that when activated they discharge repeatedly at a specific frequency. This is similar to the digital analogy given above, but rather than brain module “turning on” in response to a stimulus, the suggestion is that a circuit “resonates.”

**FIGURE 1:**
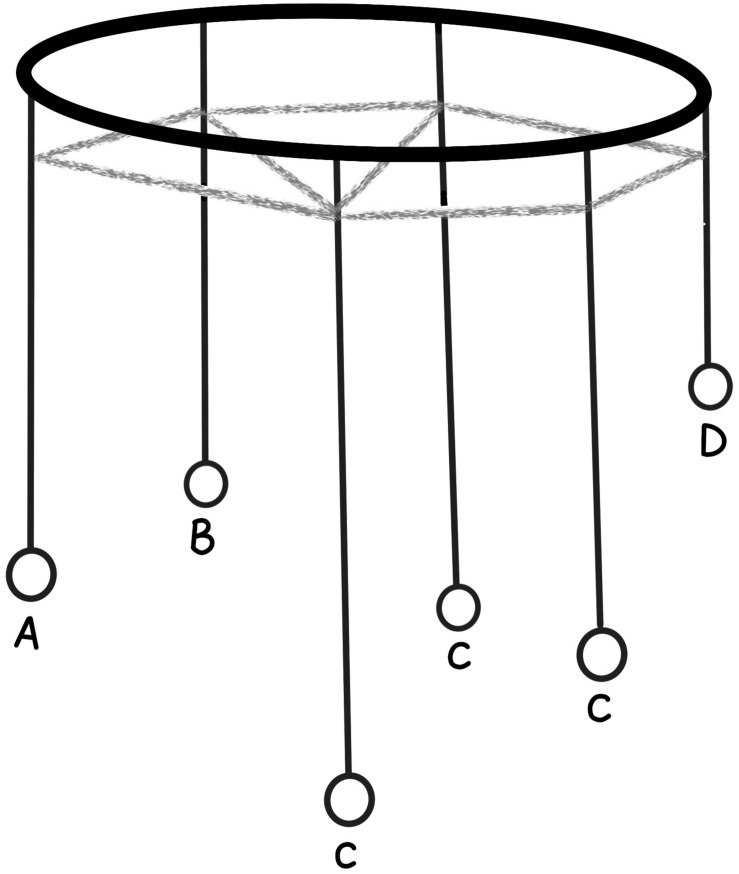
Simple analogue model with each resonant circuit represented by a pendulum. Sensory input is represented by **(A,B)**. Motion is transmitted through elastic to pendulums **(C)** with the final result expressed by the motion of pendulum **(D)**.

This can be conceptualized as a massive array of pendulums (Figure 1). Each pendulum has a length, weight and damping factor that determine its resonant frequency and how responsive it is to input. Each pendulum is then connected to an array of other pendulums by lengths of elastic string which allow each pendulum to either increase or decrease the swing of other pendulums.

At rest, each pendulum moves gently at its own resonant frequency. Pushing specific pendulums on one side of the array (sensory input) causes the amplitude of their swing to increase. This energy is then passed across the array, eventually causing the pendulums on the other side of the array to increase the amplitude of their swing (motor action). Learning is the gradual change in the length/weight of each pendulum or change in the connecting strings which change the relationship between input and output. Such a model using “coupled resonant pendulums” has already been described as providing a mathematical solution known as “quantum search” (Chen and Brylinski, 2002).

Cognitive function is therefore based on such waveform principles as resonance, harmonization, interference and temporal fluctuations rather than Boolean or Bayesian logic (Glomb et al., 2019) and our behavior would be expected to reflect these fundamental patterns (Glassman, 2000). The principle that a relatively disorganized system could create such a complex, self-organizing network through learning has already been demonstrated in a computer model (Yang et al., 2019). As a chaotic system, it would suggest that individual outputs would be highly variable but probabilistic. It would predict that while it would be difficult to predict the behavior/errors of an individual, the pattern or behavior/error in a population could be reliably predicted (Reason, 1990).

Other authors have linked such neuronal complexity to the principles of quantum physics through “quantum psychology.” The first implication is a direct implication in that as all neural circuits are constructed of matter which has quantum properties, it is inevitable that human cognition would have a probabilistic/chaotic nature rather than a deterministic/logical one. Secondly, there is a more philosophical implication that in psychology, like particle physics, “Things never ‘are’ but instead, ‘appear to be,”’ with any observation being temporary and dependent on the observer (Campagne, 2020).

Sensory input is therefore processed through a hierarchy of resonant circuits. Initial sensory input leads to resonance in low level circuits. For example in the eye, activation of rods/cones in the retina initially produces impulses in the optic nerve which then produce resonance in circuits which represent edges, colors, movement, which then move upward in the nervous system to produce resonance in circuits which represent shapes and then object and finally to circuits which represent meaning and then emotions. Correspondingly, motor action starts with a high order resonance representing a situation, which then produces resonance in progressively lower circuits representing an action, movement of limbs and finally the tension exerted by individual muscle fibers.

The initial, subconscious processing of information is not static, but a highly dynamic process, driven by a “bottom up” process by which the initial sensory inputs change the sensory process to enhance our ability to detect and make sense of the input. So, for example, if our initial visual scan detects a structure which is similar to a face, our attention will be drawn to that area and our visual pathways will auto tune to look for a familiar face. This can also be a learned process, for example, studies of experienced doctors studying photographs of patients showed that compared to novices, they took less time to extract information from the photographs, because their eyesight was directed specifically to the areas of the patients with the most relevant clinical information (Balslev et al., 2012). That is, “they knew where to look.” Again, a motor, descending process is a highly active one depending on rapid feedback from proprioceptors so that the intended action is achieved.

In the same way as above, the suggestion is that our brains are also able to apply a “top down” influence on our subconscious processing of information, so that an understanding of what we are experiencing would allow more efficient extraction of information (Miller and Cohen, 2001). This process massively reduces the quantity of information required to analyze incoming information, but also predisposes us to bias and misinterpretation. The implication is that while familiar and expected events will be perceived very quickly, the unexpected or unfamiliar will tend to be ignored or misinterpreted. That is, cognition is not viewed as making simple choices as to whether, for example, the dot on the screen has moved, or which card is correct, but rather that our environment is massively complex and constantly changing. The principle cognitive function is therefore to generate a working model of our immediate environment so that our senses can be directed to extract relevant information from specific locations.

Importantly, as this is an analog process, even with the briefest and most incomplete information would produce a pattern or resonance with that pattern either becoming stronger or changing in response to further information. For example, if we walk into a room, we would form a mental picture of the room almost as soon as we walk through the door despite the fact that our eyes may only have scanned a tiny percentage of the contents and surfaces. If we remain in the room, our own internal reality is updated by further information from our senses. This makes sense from an evolutionary process in that our brains are designed to constantly provide a workable internal model of our surroundings so that we can respond rapidly to threats or opportunities. That is, it would be better to have an analog system which rapidly suggests the presence of a predator in an uncertain situation, rather than a digital system which needs a basic set of information on which to base a logical decision. The downside is while a digital system would rarely produce errors, an analog system would be inherently less predictable.

The brain therefore is a massive collection of interconnected circuits which all resonate at different fundamental frequencies, but which all interact and influence each other to either amplify or suppress resonance in other circuits. The implication is that sensory inputs will all interact in ways that may not be logical or explicable by a logical, computational model. For example, it would be expected that some sensations like color, music, shape, and touch would share resonant frequencies and therefore be experienced by individuals as having a natural link, whereas other combinations would feel “wrong” or dissonant (Hornsby and Love, 2020). It would also be expected that apparently unconnected sensory inputs would interact. For example, it would explain why the same ice-cream tastes creamier when called Frosh compared to when it is given a harder sounding name like Frish (Doyle and Bottomley, 2011). It also implies that the investigation of human cognition in response to isolated stimuli or in a non-natural environment will inevitably produce analyses which do not reflect real world performance. Further, recent research suggests that changes to our environment are changing our cognitive structures. For example, the change from reading books which are associated with smell, tactile input and physical processes such as turning pages to the use of electronic devices will inevitably change the way we process information (Moret-Tatay and Murphy, 2019). If true, a large scale change to electronic communication could result in profound changes in our perception and interpretation of the world around us.

Cognitive overload is the default state of the brain. Our brains cannot process the quantity of information which our sensory organs generate. Therefore our cognitive circuits are designed to at a subconscious level to select information that is relevant with all other information being ignored. This highly selected information is then combined with stored patterns or schemata to generate an internal representation which is our own personal experience of the world around us. For example, although our two eyes have only small areas of color, high definition vision, our perception is that we can see everything ahead of ourselves in high resolution color. The reality is that our eyes scan backwards and forwards to pick out small details, directed to what we perceive as the key areas and our brains then construct the rest (O’Callaghan et al., 2017).

Learning is therefore the “tuning” of cognitive circuits. It follows from the above that experience would cause our cognitive circuits to resonate in response to the sensory inputs so that if we are exposed to the same stimulus repeatedly, our cognitive circuits will gradually become more receptive to the stimuli and we will recognize the pattern earlier and respond faster. Similarly, repeated practice of specific tasks will cause the relevant motor circuits to become more easily activated and to produce more accurate responses. This is exemplified in studies of expertise which emphasize prolonged, practice in real settings as the key to high performance in sport and the arts, as well as in professional practice (Ericsson et al., 1993; Cortellazzi et al., 2015). While single experiences result in some changes to our cognitive circuits, it would be expected that brief and disparate experiences would produce temporary and short lived changes, prolonged and repetitive training would result in cognitive circuits developing much more durable responses (Yang et al., 2012).

The following is written with the apparent assumption that in addition to the subconscious processes described, there is a higher center of consciousness and decision making. However, it is not the intent to suggest that such a center exists. The hypothesis here is that the brain should be considered as a single, massively interconnected analog computer and while individual anatomical sites within the brain might predominate during some functions, it suggests that consciousness and “the individual” are a function of the entire, intact brain. The implication is that the functions which have been described as “morality,” “free will,” and “personality” are, similarly, products of the entire brain.

It is crucial for any theory of cognition to be able to explain human performance within a wide range of environments and especially those outside the confines of laboratory conditions. A key principle here is that we recognize the world around us as a massively complex and rapidly changing environment which constantly threatens to overwhelm our limited cognitive power. The best examples of this is the phenomenal abilities of digital computers in highly constrained environments such as chess, where positions and moves can be represented easily in digital code. In contrast, digital computers fail in such mundane tasks such as folding a towel, because complex folds are difficult to recognize and represent in digital code (Maitin-Shepard et al., 2010).

## Evidence From Behavioral Studies

1. Where our environment changes rapidly and especially where that change resulted in an unfamiliar experience, we would expect an inability to form an internal model, leading to a failure to extract information effectively. That is, “not knowing where to look.” A subject would then experience disorientation and an inability to function. For example, seemingly competent professionals exposed to high intensity simulation would exhibit high rates of error and failure (Byrne et al., 1994; Byrne and Jones, 1997).
2. Where a subject experienced a slightly less rapid change or where the sensory input was inconsistent or only partially familiar, it would predict that even if the sensory information was incomplete, our circuits would tend to resonate in patterns which reflected our expectations. Our experiences in these situations would tend to be stereotypical and perhaps reflect personal expectations rather than reality. For example, in conditions such as twilight it would be expected that subjects would “see” ghosts or unidentified flying objects depending on which their expectations (van Prooijen et al., 2018).
3. In order to function, we require some internal model to guide our senses, so that we would tend to generate and use an internal model even if it is inconsistent with reality. From an evolutionary point of view, some error would be acceptable if it avoided paralysis. This would predict that in unusual situations, our responses would also be expected to be stereotypical or “normal,” even if the situation was highly abnormal. For example, in medical simulations, when doctors misdiagnosed the condition of the patient, their actions and perceptions appeared more consistent with their own diagnosis rather than reality (Byrne et al., 1998).
4. The suggestion here is that the process of cognition is primarily one of selecting relevant sensory inputs from the overwhelming sensory load that is available, to rapidly construct an internal reality to allow us to rapidly respond to threats or opportunities. The implication is that any loss of that filtering function with a resulting transmission of raw sensory data would be predicted to cognitively overwhelming. This explains the finding in the first real study of human efficiency in wartime, which showed that only around 25% of soldiers fired their weapons, with most paralyzed by the experience (Marshall, 2000).
5. In novel situations, as we are only capable of extracting relatively small amounts of sensory information, we would expect initial perceptions to be based on expectation and the most prominent features of the situation, with an inherent capacity for error. However, as more information was extracted, we would expect the internal mental model of a subject to develop in complexity and to reflect reality more closely, leading to the appearance of a more accurate and logical assessment of the situation. This would explain an apparent rapid (Type 1) thinking and a slower (Type II) thinking without having to posit two different systems to exist.
6. In ambiguous situations, it would also suggest that multiple circuits could be activated at the same time, effectively activating several internal models at the same time. The final conscious interpretation could then be selected by either top down choice or bottom up sensory input, explaining the visual illusions much loved by surrealist artists (Koontz and Gunderman, 2008). The implication is that the meaning of any experience must be interpreted in terms of the totality of the experience rather than as an addition of its components.
7. However, once a subject had formed a complex internal model of their surroundings it would be expected that the individual’s perception would be tuned to the expected sensory inputs required to complete what they perceive as the task in hand. Unexpected sensory inputs would then be effectively excluded from the decision making process. This would explain the “task fixation” or “tunnel vision” often demonstrated by subject in high stress situations who persist in their initial assessment of the situation despite it rapidly becoming clear that their initial assessment was incorrect (Byrne and Jones, 1997; Crane et al., 2017). It would explain why the recommended strategy to combat such behavior is the use of checklists, because they require a subject to stop and effectively “reset” their cognitive processes (Gawande, 2020).
8. In addition to the above, it would be expected that as a single, massively interconnected system of resonant circuits, the final result of the computation would be dependent on not just the summation of individual sensory inputs, but rather a highly complex, spatial, temporal and multidimensional interaction between inputs with results sometimes appearing to be chaotic. This would explain why seemingly unrelated sensory inputs, such as music, color, or taste could interact if their sensory pathways share similar resonant frequencies. It would also predict that a highly specific set of sensory inputs delivered in precisely the right temporal arrangement could trigger a powerful and seemingly unrelated response such as the experience of “Déjà vu” (Brown, 2004).
9. Perhaps the best exemplar of the need to redefine our cognitive modeling is in the area of bias, where many authors have identified that when decision making is analyzed, it demonstrates recognizable patterns of bias with what are seen as prejudicial tendencies to provide worse care for groups such as older people, women and people belonging to racial minorities (Crossley, 2003; Danziger et al., 2011). Medical decision making has also been shown to be influenced by less obvious patient characteristics such as young age, obesity, sexual orientation, personal grooming and courtesy (Hooper et al., 1982). Such bias is described in terms of faulty decision making and often linked to Type I (primitive, heuristic, rapid) with the implication that professionals need further training or to use better cognitive strategies to avoid error in the future (Croskerry et al., 2013; Hughes et al., 2020).

Although it seems undoubtedly true that such bias exists and any fair system would seek to exclude bias from any important decision making process, it ignores the evidence that such biases are largely subconscious and often in conflict with individuals’ conscious views (Chapman et al., 2013; Byrne and Tanesini, 2015). Further, it ignores evidence that decisions are influenced by factors unrelated to individual cases. For example, decisions by judges on whether to grant parole to prison inmates appeared to be decrease during each session, but returned to baseline levels after each food break (Danziger et al., 2011). The only reliable way of removing bias is to interview candidates in a way that obscures all their personal characteristics from the judges, for example, blind auditions for orchestras (Goldin and Rouse, 2000).

The ubiquity of bias is explained by animal research which shows that most decisions appear to be made after less than 100 ms and that giving a subject longer does not improve the quality of the decision (Uchida et al., 2006). More recently, and in humans, confidence in a decision is detectable via an EEG signal, even before the individual is consciously aware of that decision having been made (Lim et al., 2020). This supports the presence of a rapid, subconscious decision making system which directs behavior, with conscious thought as a *post-hoc* justification system (Patterson et al., 2012).

The evidence presented above is fragmentary and necessarily omits much evidence that is contradictory. In addition, any explanation of human cognition which is simple and easily understood must represent a gross simplification of something so complex as the human brain. The hypothesis put forward here is therefore not intended to be a literal explanation of how the brain works, but rather a different model which can be used to design alternative strategies for research.

## Implications for Cognitive Modeling

1. Post event analyses of behavior based on subject recall are likely to produce flawed conclusions as they ignore the largely subconscious nature of decision making. Therefore, research should start with independent observation and analysis of the environment to identify all possible cues used by decision makers. In particular, initial “expert panel” reviews are likely to provide highly biased and restricted decision making models.
2. Behavioral research needs to study the totality of the decision maker’s environment and that this would include much that would seem peripheral or irrelevant. Factors for consideration should include such cues as times, dates, sounds, and be specific to individual people/environments as cues may be expressed in many different ways.
3. Rather than just whether factors are present or not, their spatial, quantitative and temporal relationships may also be important as the sequence of events may be key to recognition. For example, a staff member suddenly not talking may be a highly relevant cue to them becoming stressed and an indicator that something is wrong (Nagpal et al., 2012).
4. The chaotic nature of decision making may require large datasets to reliably identify key factors for decision making. For example, to reliably identify the cues used by different individuals, it may be necessary to study multiple procedures in multiple different environments and at different times. For example, the mental workload of anesthetists was found to vary linearly over the entire 7 year training period (Byrne et al., 2013).
5. Techniques such as neural networks may be needed to identify factors which affect the behavior of participants without their being consciously aware of them. Factors such as time before lunch and time of day are factors that are likely to be important (Danziger et al., 2011).
6. Given the above factors, self-learning programs which learn from large datasets and automatically identify and link factors are more likely to provide valid solutions than those based on human interpretation of the same data.
7. Measures of success that use success/failure as outcomes are unlikely to show subtle differences in performance. In contrast, alternative outcomes such as mental workload (Davis et al., 2009), and more detailed analyses of expertise such as “optimal” performance (Cortellazzi et al., 2015) may provide better discrimination.

## Conclusion

Current theories of human cognition are inadequate to explain how humans behave in complex, high stress situations and especially where rapid decisions are made on the basis of incomplete information.

A model based on analog resonance provides a new paradigm which will support the design of research in such complex environments and should provide better insights into our subconscious decision making.

## Author Contributions

The author confirms being the sole contributor of this work and has approved it for publication.

## Conflict of Interest

The authors declare that the research was conducted in the absence of any commercial or financial relationships that could be construed as a potential conflict of interest.
